# Fluoride toothpaste, sanitary surveillance and the SUS: the case of Manaus-AM, Brazil

**DOI:** 10.11606/s1518-8787.2022056003636

**Published:** 2022-03-11

**Authors:** Mayara Costa Carneiro Ramos, Maria Augusta Bessa Rebelo, Janete Maria Rebelo Vieira, Luís Fernando Bandeira Miranda, Cinthia Pereira Machado Tabchoury, Jaime Aparecido Cury

**Affiliations:** I Universidade Federal do Amazonas Faculdade de Odontologia Programa de Pós-Graduação em Odontologia Manaus AM Brasil Universidade Federal do Amazonas. Faculdade de Odontologia. Programa de Pós-Graduação em Odontologia. Manaus, AM, Brasil; II Universidade Estadual de Campinas Faculdade de Odontologia de Piracicaba Departamento de Biociências Piracicaba SP Brasil Universidade Estadual de Campinas. Faculdade de Odontologia de Piracicaba. Departamento de Biociências. Piracicaba, SP, Brasil

**Keywords:** Toothpastes, supply & distribution, Dentifrices, chemistry, Fluorides, standards, Dental Caries, prevention & control, Dental Health Services

## Abstract

**OBJECTIVE:**

To determine the anticaries potential of toothpastes distributed by the primary health care public clinics (UBS) of Manaus, AM.

**METHODS:**

Ninety-nine tubes of toothpaste from four commercial brands were collected from October 7, 2019 to October 11, 2019 in 16 UBS. They were assigned a code by brand and source UBS. According to the information on the packaging, the four brands and their batches were formulated with sodium monofluorophosphate (Na_2_FPO_3_) and most (91%) had calcium carbonate (CaCO_3_) as an abrasive. We determined the concentrations of total fluoride (TF = TSF + InsF) and total soluble fluoride (TSF = F ions^-^ or FPO_3_^2-^), to certify whether they were in compliance with resolution ANVISA RDC No. 530 (maximum of 1,500 ppm TF) and whether they had anticaries potential (minimum of 1,000 ppm TSF). The analyses were performed with a ion- specific electrode.

**RESULTS:**

The concentrations (ppm F) of TF [mean; standard deviation (SD); n] found in toothpaste brands A (1,502.3; SD = 45.6; n = 33), B (1,135.5; SD = 52.7; n = 48) and D (936.8; SD = 20.5; N = 8) were close to those stated on the package, 1,500, 1,100 and 1,000 ppm F, respectively. In toothpaste C, we found a mean of 274.1 ppm (SD = 219.7; n = 10) of TF, which diverges from the declared concentration of 1,500 ppm F. In addition, the five tubes of lot no. 11681118 of toothpaste C did not contain fluoride. Regarding TSF, with the exception of toothpaste D (937.9; SD = 40.29), the others had a lower concentration than their respective TF.

**CONCLUSION:**

We found serious problems of quantity and quality of fluoride in toothpaste distributed by the SUS in Manaus, which shows the need for surveillance of these products and confirms the urgency of revising resolution RDC No. 530.

## INTRODUCTION

Fluoride toothpaste is recommended for preventing tooth decay because it is associated with its decline, which has been observed both in developed^1^ and developing countries, such as Brazil^2^. Toothpaste is also considered the most rational means of using fluoride because, simultaneously with the disorganization of the dental biofilm by brushing, fluoride is released into the oral cavity to interfere with the development of caries lesions or to repair existing lesions^3,4^. However, it is essential that fluoride be chemically soluble in the formulation^5^in order to be bioavailable in the mouth during brushing^6^. The effect of fluoride present in toothpaste on reduction of caries is based on evidence^7^and the minimum concentration of 1,000 ppm F is still recommended^10^. Therefore, there is a need not only for toothpaste to contain fluoride, but also to have a minimum concentration of soluble fluoride so that the population can benefit from caries control^11^.

In Brazil, 90% of the population uses toothpaste formulated with the combination of calcium carbonate (CaCO_3_) as an abrasive and sodium monofluorophosphate (Na_2_FPO_3_) as a fluoride source salt^2,12^. This type of formulation (CaCO_3_/Na_2_FPO_3_) is relatively stable since the fluoride is bound to the phosphate, therefore it does not immediately react with the Ca^++^ in the abrasive. However, due to the storage time, the MFP (FPO_3_^2-^) undergoes hydrolysis and the released fluoride ion is insolubilized by the Ca^++^ in the abrasive^4,13^. On the other hand, hydrated silica (SiO_2_) is chemically compatible with all fluoride salts (NaF, SnF_2_, AmF, Na_2_FPO_3_) used in toothpaste. However, formulations with CaCO_3_/Na_2_FPO_3_ have social impact especially for developing countries^14^ like Brazil. First, because they cost 2 to 3 times less than formulations with SiO_2_; second, and most importantly, they are widely distributed to underprivileged populations, as is done in Brazil by the Unified Health System (SUS). Thus, bids from small Brazilian manufacturers of toothpaste with CaCO_3_/Na_2_FPO_3_ win the tenders for purchases made by city halls^11^.

The quality of the fluoride in toothpaste brands available in the Brazilian market is regulated by the updated resolution RDC No. 530, of August 4, 2021^15^ of ANVISA, but like the regulations of the Southern Common Market (Mercosur)^16^ and the European Union (EU)^17^, the resolution only establishes the maximum concentration of total fluoride (TF), which is 0.15% (1,500 ppm F; mg F/kg), but not how much of this fluoride must be soluble in the formulation for anticaries efficacy^18^. As a result, in toothpaste brands sold in the Brazilian market^12^ and distributed by the SUS^11^, we found that the concentration of chemically soluble fluoride is much lower than the minimum required for anticaries potential.

The Health Department of Manaus, the capital city of Amazonas State, has the task of promoting universal access to health services according to the principles established by the SUS, which includes supplying toothpaste to underprivileged populations. Since the quality of the fluoride in toothpaste consumed by the population of Manaus is not known, the aim of this study was to evaluate whether these toothpaste brands were in compliance with resolution RDC No. 530^15^ of ANVISA in terms of TF, and whether they also had enough concentration of soluble fluoride to have anticaries potential^19^.

## METHODS

### Sampling

The toothpaste samples were obtained in primary health care public clinics (UBS) in the city of Manaus AM, Brazil, with the agreement of the Health Department (agreement no. 48/2019). We chose 16 UBS (Table 3) which had dental surgeons who are internship preceptors of the School of Dentistry at Universidade Federal do Amazonas. Ninety-nine tubes of fluoride toothpaste were collected, one tube of each batch of toothpaste available at the UBS. The toothpastes were assigned a code by brand and source UBS. The information on the packaging of the toothpaste collected is described in Table 1. The toothpaste tubes were collected between October 7, 2019 and October 11, 2019, and chemical analysis was performed between October 18, 2019 and November 7, 2019 in the laboratory of Oral Biochemistry at FOP-Unicamp, as an activity of the PROCAD/Amazon agreement 88881.200487/2018-1.

**Table 3:** Concentration (ppm F) of total soluble fluoride (TSF) found in the lots of toothpaste brands distributed at each UBS in Manaus. Amazonas State. range (Min–Max). and anticaries potential. considering the mean concentrations.

UBS	TSF (ppm F)		Anticaries potential

	Average; SD (n)	Min-Max	
Ajuricaba	536.4; 225.8 (8)	335.8–960.9	Very low to good
Arthur Virgílio	259.6; 201.0 (3)	27.4–378.0	Null to very low
Áugias Gadelha	625.8;175.4 (7)	323.9–866.0	Very low to good
Avelino Pereira	443.9; 162.2 (6)	334.4–652.9	Very low to low
Balbina Mestrinho	322.3; 205.8 (8)	87.4–738.9	Null to low
Fátima de Andrade	595.1; 263.7 (5)	327.4–950.1	Very low to good
Ivone Lima	503.8; 298.9 (9)	44.9–956.7	Null to good
Josephina de Melo	342.6; 326.0 (3)	24.4–675.9	Null to low
Mansour Bulbol	506.4;138.8 (12)	345.3–677.3	Very low to low
N-53	350.1; 16.8 (2)	338.2–362.0	Very low
N-58	487.3; 179.0 (19)	315.0–959.1	Very low to good
O-18	432.9; 236.0 (2)	266.0–599.8	Very low to low
S-04	417.0; 166.5 (6)	266.0–640.0	Very low to low
Theodomiro Garrido	599.2; 307.0 (4)	293.9–967.5	Very low to good
Vicente Palotti	185.2; 223.4 (2)	27.2–343.2	Null to very low
Vila da Prata	549.3; 357.6 (3)	340.6–962.2	Very low to good

Min: minimum value; Max: maximum value.

**Table 1:** Toothpaste (code), number of tubes/brand, lots and number (n) of tubes collected, fluoride salt, fluoride concentration (ppm F), abrasive, and expiration date stated on packaging.

Toothpastes (Code)	Number of tubes/brand	Lots (n)	Fluoride salt	ppm F	Abrasive	Expiration date
A	33	388 (4); 389 (2);	Na_2_FPO_3_	1,500	CaCO_3_	oct/20 to oct/21
		390 (5); 391 (2);				
		392 (3); 393 (2);				
		394 (5); 395 (4);				
		396(3);397(2);				
		398(1)				
B	48	99(1); 102(1);	Na_2_FPO_3_	1,100	CaCO_3_	apr/19 to oct/21
		139(5); 140(4);				
		141(6); 142(2);				
		143(2); 145(2);				
		146(1); 147(4);				
		148(4); 149(1);				
		150(4); 151(1);				
		152(2);153(2);				
		154(3); 156(2);				
		157(1)				
C	10	11681118 (5);	Na_2_FPO_3_	1,500	CaCO_3_	aug/21
		11684118 (5)				sept/21
D	8	C030(2); D031(6)	Na_2_FPO_3_	1,000	Silica	Feb. 2021

Na_2_FPO_3_ = sodium monofluorophosphate; CaCO_3_ = calcium carbonate.

### Determining Fluoride Concentration

Fluoride concentration was measured with a ion-specific electrode through the direct technique, using a validated methodology^20,21^. The concentrations (ppm F = mg F/Kg) of total fluoride (TF = TSF + InsF), total soluble fluoride (TSF = fluoride as FPO_3_^2-^ + IF) and ionic fluoride (IF) were determined and the concentration of fluoride as an FPO_3_^2-^ ion (= TSF - IF) and as insoluble fluoride (InsF = TF found - TSF found) were estimated. Summarily, between 90 and 110 mg of toothpaste from each tube were weighed and homogenized in 10 mL of purified water. Duplicates of 0.25 mL of the toothpaste suspension were transferred to tubes marked TF. The remainder of the toothpaste suspension was centrifuged and 0.25 mL duplicates of the supernatant were transferred to tubes marked TSF and IF. We added 0.25 mL of 2 M HCl to the TF and TSF tubes, and after one hour at 45°C, the samples were neutralized with 0.50 mL of 1 M NaOH and buffered with 1.0 mL of TISAB II (1 M acetate buffer, pH 5.0, containing 1 M NaCl and 0.4% CDTA). We added sequentially to the IF tubes 0.50 mL of 1 M NaOH, 1.0 mL of TISAB II and 0.25 mL of 2 M HCl.

The analyses were performed with a fluoride ion-specific electrode (Thermo Scientific Orion 96-09, Orion Research, Cambridge, MA, USA) coupled to an ion analyzer (Thermo Scientific Orion Star A214, Orion Research). The electrode was calibrated in triplicate, with standard fluoride solutions of 0.0625 to 4 µg F/mL prepared in 0.25 M HCl, 0.25 M NaOH and TISAB II 50% (v/v). The logarithm data of the fluoride concentrations of the standards and the respective mV values were analyzed by linear regression, using Microsoft Excel software (Microsoft^®^, Redmond, USA). The mean linear regression coefficient obtained was R^2^ = 0.9998 (n = 14) and the mathematical regression equation was used to estimate the fluoride concentration in each analytical mixture (µg F/mL). The average percentage of the coefficient of variation of duplicates was less than 2.5%. The fluoride concentration in each brand of toothpaste was estimated based on the weight of the toothpaste in the analysis tube and expressed in ppm of F (mg F/Kg).

### Data Analysis

The means of the dosage duplicates were calculated and used to estimate the mean and standard deviation of the TF, TSF and InsF concentrations for each brand of toothpaste, using the Microsoft Excel software (Microsoft^®^). We estimated the anticaries potential of the concentration of TSF in the distributed toothpastes^19^.

## RESULTS

Table 1 shows that of the 99 toothpastes collected, 33 tubes (12 lots) were of Brand A, 48 (19 lots) of brand B, 10 (two lots) of brand C and 8 (two lots) of brand D. All were formulated with sodium monofluorophosphate (Na_2_FPO_3_) at concentrations between 1,000 and 1,500 ppm F. With the exception of toothpaste D, which was formulated with SiO_2_, the toothpastes contained CaCO_3_ as an abrasive. With the exception of sample lot 099 of brand B, all toothpastes collected were within the specified expiration date. Toothpaste samples C (n = 10) and D (N = 8) were not available at all 16 UBS. The fact that there were only two batches of toothpastes C and D reflected in the results for fluoride concentration of the products distributed by the UBS and districts.

The figure shows the fluoride concentrations found in all tubes of each brand of toothpaste distributed at the UBS. With the exception of toothpaste C, the mean concentration (ppm F) (±SD; n) of TF found in brands D (936.8 ± 20.5; 8), A (1,502.3 ± 45.6; 33) and B (1,135.5 ± 52.7; 48) was very close to the manufacturers’ specifications. Regarding the analysis of toothpaste C, besides the concentration of TF being much lower than the 1,500 ppm F specified (274.1 ± 219.7; 10), the SD was extremely high (80% of the mean), reflecting the difference in concentration of the two batches of this product.

**Figure:**
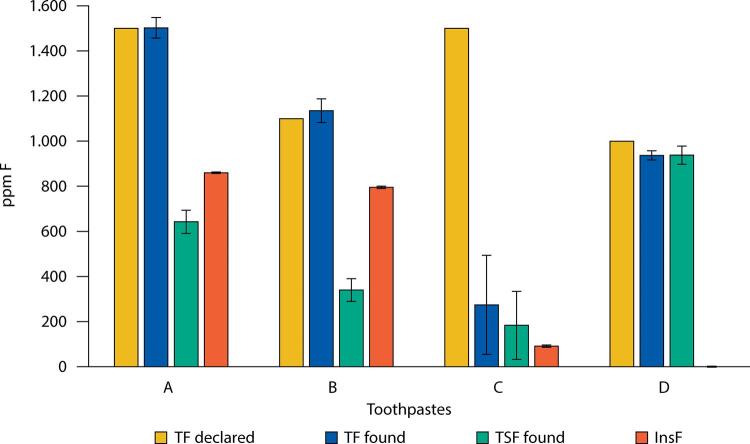
Concentration (ppm F; mg F/kg) of total fluoride (TF) declared on the package, mean and SD (bars) of the concentrations of TF found, total soluble fluoride (TSF), and insoluble fluoride (insF) in toothpaste brands distributed by the UBS of Manaus, AM.

As for the values (mean ± SD; n) of TSF found (figure and Table 2), only toothpaste D exhibited a concentration (937.9 ± 40.3; 8) close to the TF found; insoluble fluoride (InsF) was not found in this product (Figure). In the other brands, much of the TF found (figure) was not soluble as TSF (FPO_3_^2-^ + F^-^), with 36, 57 and 70% of InsF in toothpastes C, A and B, respectively. The case of toothpaste C is totally anomalous.

**Table 2:** Concentration (ppm F) of total soluble fluoride (TSF) found in the lots of toothpaste brands distributed at the UBS in Manaus. Amazonas State. range (Min–Max). and anticaries potential. considering the mean concentrations.

Toothpaste (Code)	Lots	TSF (ppm F)		Anticaries potential

		Average; SD (n)	Min-Max	
A	All (388 to 398)	642.4; 51.3 (33)	525.4–777.0	Low
B	All (99 to 157)	339.9; 50.5 (48)	108.9–427.7	Very low
C	11681118	42.3; 28.4 (5)	24.4–87.4	Null
	11684118	324.1; 18.6 (5)	293.0–345.1	Very low
D	All (C030 and D031)	937.9; 40.3 (8)	866.0–967.5	Good

Min: minimum value; Max: maximum value.

As for the anticaries potential^19^ of the toothpaste brands distributed in Manaus, Table 2 took into account the average TSF concentration of all toothpaste lots of each brand, Table 3 the average concentration of brands that were being distributed at each UBS, and Table 4 the location of the UBS according to the health district of Manaus. According to Table 2, the toothpaste brands analyzed can be grouped into the following descending order of anticaries potential (Good to Null): D > A > B = C (lot 11684118) > C (lot 11681118).

**Table 4:** Concentration (ppm F) of total soluble fluoride (TSF) found in toothpaste brands distributed at the Health Districts of Manaus. Amazonas State. and anticaries potential. considering the interval of concentrations.

Districts	TSF (ppm F)		Anticaries potential

	Average; SD (n)	Min-Max	
North	465.3; 315.3 (44)	27.4–959.1	Null to good
South	439.1; 253.3 (12)	27.2–967.4	Null to good
East	457.0; 256.6 (18)	24.4–956.7	Null to good
West	515.3; 193.8 (25)	266.0–962.2	Very low to good

Table 3 shows that no UBS were distributing toothpaste with exclusively good or null anticaries potential, the best result ranging from very low to good in six of the 16 UBS, which was reflected in the assisted populations living in the four health districts of Manaus (Table 4). Thus, only the Western District distributed toothpaste with very low to good anticaries potential, while the other districts had toothpastes with null to good potential.

## DISCUSSION

The WHO has recommended affordable fluoride toothpaste for populations as one of the strategies for reducing tooth decay^14^ since this disease affects more than 2.5 billion people around the world^22^. Toothpaste brands with CaCO_3_ as the abrasive has lower cost (2-3x<) than those formulated with SiO_2_^3,14^, and there are fluoride toothpastes in the Brazilian market formulated with CaCO_3_/Na_2_FPO_3_ of acceptable quality in terms of the concentration of potentially active fluoride against caries^12,13,23^. Although toothpaste based on CaCO_3_/Na_2_FPO_3_ is affordable in Brazil (R$ 0.028/g), 50% of the population would have to work approximately one hour a day just to buy a 90 g tube of toothpaste. Therefore, due to social inequality in Brazil, the Brazilian government runs a preventive action program for SUS users as an action to promote oral health^24^. Toothpaste is bought through a public bidding process and the winning firms are usually from local manufacturers of toothpaste based on CaCO_3_/Na_2_FPO_3_^11^. The quality of this toothpaste in terms of anticaries potential could be guaranteed not only in Brazil, but worldwide, if there were support for government regulations on the sale of toothpaste^18^.

Our results showed that the total fluoride concentration (TF) found in the toothpaste distributed by the SUS in Manaus (Figure) matches the specifications on the packaging of three of the four brands analyzed (Table 1). The exception was toothpaste C, in which we expected to find 1,500 ppm TF (Table 1), but the value found was 82% lower. In addition to the low mean value found, of 274.1 ppm TF in this product, another relevant aspect was the high standard deviation from the mean (80%) of the 10 tubes analyzed. This is easily explained by the difference in TF concentration found between the two batches of this brand. While in the 5 tubes of toothpaste C of batch 11684118, we found a mean of 479.6 ppm F TF, in batch 11681118 we only found 68.4 ppm F TF. Thus, the toothpaste of batch 11684118 had an amount of Na_2_FPO_3_ three times lower than stated on the packaging, while lot 11684118 did not contain any fluoride at all. The mean value of 68.4 ppm F TF in the tubes of this batch can be attributed to the residual fluoride of the ingredients used in its formulation. Thus, this toothpaste violates Brazilian^15^ and Mercosur regulations^16^ since it was not formulated as stated on the labeling. It is also admissible that the supplier of the product for the SUS in Manaus failed to comply with the tender because the standardized specification for such products is that toothpaste must contain fluoride in the concentration of 1,000 to 1,500 ppm F. This blatant aberration of not finding the stated TF concentration in toothpaste is rare in our 40 years evaluating toothpaste worldwide, and it has only been observed in two brands of toothpaste from China, one being sold in Chile^25^ and the other in Peru^19^. On the other hand, the most important aspect in terms of health is how much of the TF in toothpaste is chemically soluble to have anticaries potential^3-5^.

Thus, of the four brands of toothpaste analyzed that were being distributed by the SUS in Manaus, only brand D would have a good anticaries potential (Table 2) because it exhibited an average of 937.9 ppm F of total soluble fluoride (TSF), very close to the minimum value of 1,000 ppm F necessary for anticaries efficacy^4^. In addition, all fluoride in this toothpaste is soluble (figure) since it was formulated with SiO_2_, an abrasive compatible with all fluoride salts, including Na_2_FPO_3_, used in this formulation (Table 1). On the other hand, the concentration of TSF found in the other brands of toothpaste was very low. The low concentration of TSF found in brands A, B and C (Table 2) is explained not only because they contain CaCO_3_ as an abrasive (Table 1), since it inactivates fluoride^3,4^, but also by the fact that, with the exception of brand A, these toothpaste brands were formulated with less than 1,500 ppm TF.

As 57% of the TF in brand A was insoluble (Figure), 642.3 ppm remained as TSF, which gives it low anticaries potential (Table 2). Brand B, on the other hand, in addition to being formulated with 1,100 ppm F TF (Table 1), has 70% insoluble fluoride, meaning only 339.9 ppm of TSF, which gives it very low anticaries potential. Compared to product A, the higher percentage of InsF in B can be explained by the fact that their expiration date is close (Table 1), including batch 99 toothpaste of Brand B, distributed at UBS Balbina Mestrinho, which had expired (April 2019) six months before collection. In the toothpaste of this batch, of the 1,100 ppm of TF added, only 108.9 ppm were soluble (Table 2), that is, 90% of its TF of was insoluble, therefore not exhibiting any anticaries effect. This result of decreased concentration of TSF in toothpaste brands formulated with CaCO_3_/Na_2_FPO_3_ according to the time elapsed since manufacture is well known in the literature^13^, but the percentage values found in this study were much higher.

On the other hand, the case of toothpaste C is totally atypical because the very low average concentration of 42.3 ppm of TSF found in the tubes of lot 11681118 is due to the fact that it has not even been fluoridated, which gives it zero anticaries potential. In toothpastes C of batch 11684118, a mean of 324.1 ppm of TSF was found, since 32% of the 479.7 ppm of the TF found was insoluble, which gives it a very low anticaries potential (Table 2).

Considering that of the 99 toothpaste tubes analyzed, only the eight of brand D can be considered to have good anticaries potential (Table 2), which represents only 8% of the sample, it is very unlikely that any UBS of Manaus was distributing only good quality toothpaste to its users, as the data in Table 3 show. Thus, if at one end there were people receiving toothpaste with good anticaries potential, in the case of brand D, at the other there were people receiving toothpaste with no protection against caries (Null), as was the case of batch 11681118 of toothpaste C. Similarly, no health district of the city of Manaus had benefited from the quality of fluoride toothpaste distributed, as shown in Table 4.

This problem of low-quality fluoride toothpaste is not exclusive to Brazil and has been reported in other countries^26,27^. With the exception of the regulations of the US^28^ and Madagascar^29^, most of the world’s regulations^16,17^ do not set forth that toothpaste have soluble fluoride for anticaries efficacy, but the FDI has recently signaled that this needs to change^30^.

Our results corroborate previous publications^11,18^and clearly show the need for a revision of the Brazilian regulation ANVISA RDC No. 530^15^ to ensure that the population, especially the one assisted by the SUS, receives fluoride toothpaste with a minimum concentration of soluble fluoride with anticaries potential. As Brazil is part of the Mercosur, there may be multilateral interests that need to be agreed between the countries of this common market for changes in legislation. While this does not happen, the alternative would be for SUS managers to draft tender notices with the following terms^11^:

1. Toothpaste must contain no more than 1,500 ppm (mg/kg) of total fluoride (TF);
2. At least 1,000 ppm of TF must be chemically soluble (TSF) in a fresh (newly manufactured) sample;
3. Toothpaste must keep at least 800 ppm of chemically soluble fluoride (TSF) for 2 years after manufacture.

The reason for the maximum amount of 1,500 ppm of TF is product safety for free sale, the requirement of 1,000 ppm of soluble fluoride aims to ensure a minimum anticaries effect, and 800 soluble ppm for 2 years aims to ensure a feasible concentration^13^ even for small Brazilian toothpaste manufacturers, so as not to sideline them from the competition with multinational companies, considering that they are the ones that win the bids made by the Brazilian Public Health System^11^.

## CONCLUSION

Considering the serious problems of quantity and quality of fluoride in toothpaste brands distributed by the SUS in Manaus, the main conclusion of this article is that a revision of ANVISA RDC No. 530 and world regulations would ensure that not only Brazilians, but also citizens of other developing countries, no longer run the risk of using fluoride toothpaste that is potentially ineffective in controlling dental caries.
