# Supplementary material for: Fluoride toothpaste, sanitary surveillance and the SUS: the case of Manaus-AM, Brazil
Source: Rev Saude Publica. 2022 Mar 11;56:9. doi: 10.11606/s1518-8787.2022056003636 (PMC8926396; doi:10.11606/s1518-8787.2022056003636)
Supplement: Translation

# Translation: Dentifrício fluoretado, vigilância sanitária e o SUS: o caso de Manaus-AM

**Authors:** Mayara Costa Carneiro Ramos, Maria Augusta Bessa Rebelo, Janete Maria Rebelo Vieira, Luís Fernando Bandeira Miranda, Cinthia Pereira Machado Tabchoury, Jaime Aparecido Cury

**Affiliations:** I Brasil Universidade Federal do Amazonas. Faculdade de Odontologia. Programa de Pós-Graduação em Odontologia. Manaus, AM, Brasil; II Brasil Universidade Estadual de Campinas. Faculdade de Odontologia de Piracicaba. Departamento de Biociências. Piracicaba, SP, Brasil

**Keywords:** Cremes Dentais, provisão & distribuição, Dentifrícios, química, Fluoretos, normas, Cárie Dentária, prevenção & controle, Serviços de Saúde Bucal

## Abstract

**OBJETIVO:**

Determinar o potencial anticárie dos dentifrícios distribuídos pelas unidades básicas de saúde (UBS) de Manaus-AM.

**MÉTODOS:**

Noventa e nove bisnagas de dentifrícios de quatro marcas comerciais foram coletadas de 7 de outubro de 2019 a 11 de outubro de 2019 em 16 UBS, que foram codificados por marca e UBS de origem. De acordo com a embalagem, os dentifrícios das quatro marcas e seus lotes foram formulados com monofluorofosfato de sódio (Na_2_FPO_3_) e a maioria (91%) tinha carbonato de cálcio (CaCO_3_) como abrasivo. Foram determinadas as concentrações de fluoreto total (FT = FST + Fins) e de fluoreto solúvel total (FST = íons F^-^ ou FPO_3_^2-^), para certificar se atendiam à resolução ANVISA RDC Nº 530 (máximo de 1.500 ppm de FT) e se tinham potencial anticárie (mínimo de 1.000 ppm de FST). As análises foram feitas com eletrodo íon específico.

**RESULTADOS:**

As concentrações (ppm F) de FT [média; desvio padrão (DP); n] encontradas nos dentifrícios A (1.502,3; DP = 45,6; n = 33), B (1.135,5; DP = 52,7; n = 48) e D (936,8; DP = 20,5; n = 8) foram próximas ao descrito na embalagem, 1.500, 1.100 e 1.000 ppm F, respectivamente. No dentifrício C, foi encontrada média de 274,1 ppm (DP = 219,7; n = 10) de FT, divergindo da concentração declarada de 1.500 ppm F. Em acréscimo, as cinco bisnagas do lote n^o^ 11681118 do dentifrício C não foram fluoretadas. Quanto ao FST, à exceção do dentifrício D (937,9; DP = 40,29), os demais apresentaram concentração inferior ao seu respectivo FT.

**CONCLUSÃO:**

Observou-se problemas graves de quantidade e qualidade do fluoreto nos dentifrícios distribuídos pelo SUS em Manaus, mostrando a necessidade de vigilância desses produtos e ratificando a urgência da revisão da resolução ANVISA RDC Nº 530.

## INTRODUÇÃO

Dentifrício fluoretado é recomendado no combate à cárie dentária por estar relacionado ao seu declínio, o qual foi observado tanto em países desenvolvidos^1^ como em desenvolvimento, como o Brasil^2^. Dentifrício é também considerado o meio mais racional de uso de fluoreto, porque simultaneamente à desorganização do biofilme dental pela escovação, o fluoreto é liberado na cavidade bucal para interferir no desenvolvimento de lesões de cárie ou reparar as lesões existentes^3,4^. Entretanto, para tal é imprescindível que o fluoreto esteja quimicamente solúvel na formulação^5^para ser biodisponibilizado na boca durante a escovação^6^. O efeito do fluoreto presente nos dentifrícios na redução de cárie está baseado em evidências^7^e a concentração de no mínimo 1.000 ppm F continua sendo recomendada^10^. Portanto, há necessidade de não só o dentifrício ser fluoretado como também possuir uma concentração mínima de fluoreto solúvel para que a população seja beneficiada em termos de controle de cárie^11^.

No Brasil, 90% da população utiliza dentifrícios formulados com a combinação de carbonato de cálcio (CaCO_3_) como abrasivo e monofluorofosfato de sódio (Na_2_FPO_3_) como sal de fonte de fluoreto^2,12^. Esse tipo de formulação (CaCO_3_/Na_2_FPO_3_) apresenta relativa estabilidade, pois o fluoreto está ligado ao fosfato, logo não reage imediatamente com o Ca^++^ do abrasivo. No entanto, em razão do tempo de armazenamento, o íon MFP (FPO_3_^2-^) sofre hidrólise e o íon fluoreto liberado é insolubilizado pelo Ca^++^ do abrasivo^4,13^. Por outro lado, a sílica hidratada (SiO_2_) é quimicamente compatível com todos os sais de fluoreto (NaF, SnF_2_, AmF, Na_2_FPO_3_) usados em dentifrícios. Entretanto, formulações com CaCO_3_/Na_2_FPO_3_ têm impacto social principalmente para países em desenvolvimento^14^ como o Brasil. Primeiro porque custam 2-3 vezes menos que as formulações com SiO_2_; segundo e mais importante, são amplamente distribuídos para populações com menor condição socioeconômica, como é feito no Brasil pelo Sistema Único de Saúde (SUS). Assim, propostas de pequenos produtores brasileiros de dentifrício com CaCO_3_/Na_2_FPO_3_ vencem os processos de licitação de compras realizadas pelas prefeituras^11^.

A qualidade do fluoreto dos dentifrícios disponíveis no mercado brasileiro é regulamentada pela atualizada resolução ANVISA RDC Nº 530, de 4 de agosto de 2021^15^, mas, à semelhança das regulamentações do Mercado Comum do Sul (Mercosul)^16^ e da União Europeia (EUR)^17^, a resolução apenas estabelece a concentração máxima de fluoreto total (FT), que é 0,15% (1.500 ppm F; mg F/kg), mas não o quanto desse fluoreto deve estar solúvel na formulação para ter efeito anticárie^18^. Assim, tanto nos dentifrícios à venda no mercado brasileiro^12^ como naqueles que são distribuídos pelo SUS^11^, encontra-se concentração de fluoreto quimicamente solúvel muito inferior ao mínimo necessário para ter potencial anticárie.

A Secretaria Municipal de Saúde de Manaus, capital do estado do Amazonas, possui a função de promover o acesso universal aos serviços de saúde de acordo com os princípios estabelecidos no SUS, o que inclui a oferta de dentifrícios à população menos favorecida. Como a qualidade do fluoreto dos dentifrícios consumidos pela população manauara não é conhecida, o objetivo desse trabalho foi avaliar se esses dentifrícios atendiam à resolução ANVISA RDC Nº 530^15^ em termos de FT e se também tinham concentração de fluoreto solúvel para ter potencial anticárie^19^.

## MÉTODOS

### Amostragem

As amostras de dentifrícios foram obtidas em Unidades Básicas de Saúde (UBS) da cidade de Manaus, AM, Brasil, após concordância da Secretaria de Saúde (termo de compromisso nº 48/2019). Foram escolhidas 16 UBS (Tabela 3), nas quais havia cirurgiões-dentistas preceptores do estágio curricular da Faculdade de Odontologia da Universidade Federal do Amazonas. Noventa e nove bisnagas de dentifrícios fluoretados foram coletadas, sendo uma bisnaga de cada lote de dentifrício disponível nas UBS. Os dentifrícios foram codificados por marca e UBS de origem. As informações sobre os dentifrícios coletados encontradas nas embalagens estão descritas na Tabela 1. Os dentifrícios foram coletados entre o dia 7 de outubro de 2019 e 11 de outubro de 2019, e realizou-se as análises químicas entre os dias 18 de outubro de 2019 e 7 de novembro de 2019, no laboratório de Bioquímica Oral da FOP-Unicamp, como atividade do convênio PROCAD/Amazônia 88881.200487/2018-1.

**Tabela 3:** Concentração (ppm F) de fluoreto solúvel total (FST) encontrada nos lotes de dentifrícios distribuídos em cada UBS de Manaus, AM, intervalo (Min–Max) e potencial anticárie, considerando a média das concentrações.

UBS	FST (ppm F)		Potencial anticárie

	Média; DP (n)	Min–Max	
Ajuricaba	536,4; 225,8 (8)	335,8–960,9	Muito baixo a bom
Arthur Virgílio	259,6; 201,0 (3)	27,4–378,0	Nulo a muito baixo
Áugias Gadelha	625,8;175,4 (7)	323,9–866,0	Muito baixo a bom
Avelino Pereira	443,9; 162,2 (6)	334,4–652,9	Muito baixo a baixo
Balbina Mestrinho	322,3; 205,8 (8)	87,4–738,9	Nulo a baixo
Fátima de Andrade	595,1; 263,7 (5)	327,4–950,1	Muito baixo a bom
Ivone Lima	503,8; 298,9 (9)	44,9–956,7	Nulo a bom
Josephina de Melo	342,6; 326,0 (3)	24,4–675,9	Nulo a baixo
Mansour Bulbol	506,4;138,8 (12)	345,3–677,3	Muito baixo a baixo
N-53	350,1; 16,8 (2)	338,2–362,0	Muito baixo
N-58	487,3; 179,0 (19)	315,0–959,1	Muito baixo a bom
O-18	432,9; 236,0 (2)	266,0–599,8	Muito baixo a baixo
S-04	417,0; 166,5 (6)	266,0–640,0	Muito baixo a baixo
Theodomiro Garrido	599,2; 307,0 (4)	293,9–967,5	Muito baixo a bom
Vicente Palotti	185,2; 223,4 (2)	27,2–343,2	Nulo a muito baixo
Vila da Prata	549,3; 357,6 (3)	340,6–962,2	Muito baixo a bom

Min: valor mínimo; Max: valor máximo.

**Tabela 1:** Dentifrícios (código), quantidade de tubos/marca, lotes e quantidade (n) de tubos coletados, sal de fluoreto, concentração de fluoreto (ppm F), abrasivo e validade declaradas nas embalagens.

Dentifrícios (Código)	Quantidade tubos/marca	Lotes (n)	Sal de fluoreto	ppm F	Abrasivo	Validade
A	33	388 (4); 389 (2);	Na_2_FPO_3_	1.500	CaCO_3_	out/20 a out/21
		390 (5); 391 (2);				
		392 (3); 393 (2);				
		394 (5); 395 (4);				
		396(3);397(2);				
		398(1)				
B	48	99(1); 102(1);	Na_2_FPO_3_	1.100	CaCO_3_	abr/19 a out/21
		139(5); 140(4);				
		141(6); 142(2);				
		143(2); 145(2);				
		146(1); 147(4);				
		148(4); 149(1);				
		150(4); 151(1);				
		152(2);153(2);				
		154(3); 156(2);				
		157(1)				
C	10	11681118 (5);	Na_2_FPO_3_	1.500	CaCO_3_	ago/21
		11684118 (5)				set/21
D	8	C030(2); D031(6)	Na_2_FPO_3_	1.000	Sílica	fev/21

Na_2_FPO_3_ = monofluorofosfato de sódio; CaCO_3_ = carbonato de cálcio.

### Determinação da Concentração de Fluoreto

Foi realizada com eletrodo íon específico pela técnica direta, usando metodologia validada^20,21^. As concentrações (ppm F = mg F/Kg) de fluoreto total (FT = FST + Fins), fluoreto solúvel total (FST = fluoreto como íon FPO_3_^2-^ + FI) e fluoreto iônico (FI) foram determinadas, sendo que a concentração de fluoreto como íon FPO_3_^2-^ (= FST – FI) e como fluoreto insolúvel (Fins = FT encontrado – FST encontrado) foram calculadas. Sumariamente, entre 90 e 110 mg de dentifrício de cada tubo foi pesado e homogeneizado em 10 mL de água purificada. Duplicatas de 0,25 mL da suspensão foram transferidas para tubos marcados FT. O restante da suspensão foi centrifugado e duplicatas de 0,25 mL do sobrenadante foram transferidas para tubos marcados FST e FI. Aos tubos FT e FST, 0,25 mL de HCl 2 M foram acrescentados e após uma hora a 45°C, as amostras foram neutralizadas com 0,50 mL de NaOH 1 M e tamponadas com 1,0 mL de TISAB II (tampão acetato 1 M, pH 5,0, contendo NaCl 1 M e CDTA 0,4%). Aos tubos FI foram adicionadas sequencialmente 0,50 mL de NaOH 1 M, 1,0 mL de TISAB II e 0,25 mL de HCl 2 M.

As análises foram feitas com eletrodo íon específico para fluoreto (Thermo Scientific Orion 96-09, Orion Research, Cambridge, MA, EUA) acoplado a um analisador de íons (Thermo Scientific Orion Star A214, Orion Research). O eletrodo foi calibrado, em triplicata, com soluções padrões de fluoreto de 0,0625 a 4 μg F/mL preparados em HCl 0,25 M, NaOH 0,25 M e TISAB II 50% (v/v). Os dados do logaritmo das concentrações de fluoreto dos padrões e os respectivos valores de mV foram analisados por regressão linear, utilizando o software Microsoft Excel (Microsoft^®^, Redmond, EUA). O coeficiente de regressão linear médio obtido foi de R^2^ = 0,9998 (n = 14) e a equação da regressão matemática foi utilizada para estimar a concentração de fluoreto em cada mistura analítica (µg F/mL). A média da porcentagem do coeficiente de variação das duplicatas foi menor que 2,5%. A concentração de fluoreto em cada marca de dentifrício foi calculada com base no peso de dentifrício no tubo de análise e expressa em ppm de F (mg F/Kg).

### Análise de Dados

As médias das duplicatas de dosagem foram calculadas e utilizadas para calcular a média e o desvio padrão das concentrações de FT, FST e Fins para cada marca de dentifrício, utilizando o software Microsoft Excel (Microsoft^®^). O potencial anticárie da concentração de FST nos dentifrícios distribuídos foi estimado^19^.

## RESULTADOS

A Tabela 1 mostra que, dos 99 dentifrícios coletados, 33 tubos (12 lotes) eram da marca A, 48 (19 lotes) da marca B, 10 (dois lotes) da marca C e 8 (dois lotes) da marca D. Todos foram formulados com monofluorofosfato de sódio (Na_2_FPO_3_) nas concentrações entre 1.000 e 1.500 ppm F. Com exceção do dentifrício D, que foi formulado com SiO_2_, os dentifrícios continham CaCO_3_ como abrasivo. Com exceção da amostra lote 099 da marca B, todos os dentifrícios coletados estavam dentro do prazo de validade especificado. As amostras dos dentifrícios C (n = 10) e D (n = 8) não estavam disponíveis em todas as 16 UBS. O fato de haver apenas dois lotes dos dentifrícios C e D refletiu nos resultados encontrados de concentração de fluoreto dos produtos distribuídos pelas UBS e distritos.

A Figura mostra as concentrações de fluoreto encontradas em todos os tubos de cada marca de dentifrício distribuídos nas UBS. À exceção do dentifrício C, a concentração (ppm F) média (±dp;n) de FT encontrada nos dentifrícios D (936,8 ± 20,5;8), A (1.502,3 ± 45,6; 33) e B (1.135,5 ± 52,7;48) foi muito próxima à declarada pelos fabricantes. Quanto à análise do dentifrício C, além da concentração de FT estar muito abaixo dos 1.500 ppm F declarados (274,1 ± 219,7;10), o dp foi extremamente alto (80% da média), refletindo a diferença de concentração dos dois lotes desse produto.

**Figura:** Concentração (ppm F; mg F/kg) de fluoreto total (FT) declarada na embalagem, média e dp (barras) das concentrações encontradas de FT, fluoreto solúvel total (FST), e de fluoreto insolúvel (Fins) nos dentifrícios distribuídos pelas UBS de Manaus, AM.

Quanto aos valores (média ± dp;n) de FST encontrados (Figura e Tabela 2), apenas o dentifrício D apresentou concentração (937,9 ± 40,3;8) próxima do FT encontrado; fluoreto insolúvel (Fins) não foi encontrado nesse produto (Figura). Nos demais dentifrícios, grande parte do FT encontrado (Figura) não estava solúvel como FST (íons FPO_3_^2-^ + F^-^), havendo 36, 57 e 70% de Fins nos dentifrícios C, A e B, respectivamente. O caso do dentifrício C é totalmente anômalo.

**Tabela 2:** Concentração (ppm F) de fluoreto solúvel total (FST) encontrada nos lotes de dentifrícios distribuídos nas UBS de Manaus, AM, intervalo (Min–Max) e potencial anticárie, considerando a média das concentrações.

Dentifrícios (Código)	Lotes	FST (ppm F)		Potencial anticárie

		média; DP (n)	Min–Max	
A	Todos (388 a 398)	642,4; 51,3 (33)	525,4–777,0	Baixo
B	Todos (99 a 157)	339,9; 50,5 (48)	108,9–427,7	Muito baixo
C	11681118	42,3; 28,4 (5)	24,4–87,4	Nulo
	11684118	324,1; 18,6 (5)	293,0–345,1	Muito baixo
D	Todos (C030 e D031)	937,9; 40,3 (8)	866,0–967,5	Bom

Min: valor mínimo; Max: valor máximo.

Quanto ao potencial anticárie^19^ dos dentifrícios distribuídos em Manaus, a Tabela 2 levou em consideração a média da concentração de FST de todos os dentifrícios de cada marca, a Tabela 3 da concentração daqueles que estavam sendo distribuídos em cada UBS e a Tabela 4, a localização das UBS pelos Distritos de Saúde de Manaus. De acordo com a Tabela 2, os dentifrícios analisados podem ser classificados na seguinte ordem decrescente de potencial anticárie (Bom a Nulo): D > A > B = C (lote 11684118) > C (lote 11681118).

**Tabela 4:** Concentração (ppm F) de fluoreto solúvel total (FST) encontrada nos dentifrícios distribuídos nos Distritos de Saúde de Manaus, AM, e potencial anticárie, considerando o intervalo das concentrações.

Distritos	FST (ppm F)		Potencial anticárie

	Média; DP (n)	Min–Max	
Norte	465,3; 315,3 (44)	27,4–959,1	Nulo a bom
Sul	439,1; 253,3 (12)	27,2–967,4	Nulo a bom
Leste	457,0; 256,6 (18)	24,4–956,7	Nulo a bom
Oeste	515,3; 193,8 (25)	266,0–962,2	Muito baixo a bom

A Tabela 3 mostra que não havia nenhuma UBS distribuindo dentifrício de potencial anticárie exclusivamente Bom ou Nulo, sendo o melhor resultado de Muito Baixo a Bom em seis das 16 UBS, o que se refletiu nas populações beneficiadas vivendo nos quatro distritos de saúde de Manaus (Tabela 4). Assim, somente no distrito Oeste estavam sendo distribuídos dentifrícios de Muito Baixo a Bom potencial anticárie, enquanto nos demais foi de Nulo a Bom.

## DISCUSSÃO

A OMS tem recomendado dentifrício fluoretado de custo acessível às populações como uma das estratégias para redução de cárie dentária^14^, uma vez que essa doença acomete mais de 2,5 bilhões de pessoas ao redor do mundo^22^. Dentifrícios com CaCO_3_ como abrasivo são de menor custo (2-3x<) que os formulados com SiO_2_^3,14^ e, no mercado brasileiro, há dentifrícios fluoretados formulados com CaCO_3_/Na_2_FPO_3_ de qualidade aceitável quanto à concentração de fluoreto potencialmente ativo contra cárie^12,13,23^. Embora no Brasil o custo de um creme dental à base de CaCO_3_/Na_2_FPO_3_ seja acessível (R$ 0,028/g), 50% da população teria que trabalhar aproximadamente uma hora por dia só para comprar um tubo de 90 g de creme dental. Assim, devido à desigualdade social no Brasil, o governo brasileiro mantém um programa de ações preventivas aos usuários do SUS, como ação de promoção de saúde bucal^24^. Os dentifrícios são adquiridos por um processo de licitação pública e as firmas vencedoras geralmente são de produtores locais de dentifrícios à base de CaCO_3_/Na_2_FPO_3_^11^. A qualidade desses dentifrícios em termos de potencial anticárie poderia ser garantida não só no Brasil, como no mundo inteiro, se houvesse respaldo nas regulamentações governamentais sobre a venda de dentifrícios^18^.

Nossos resultados mostraram que a concentração de fluoreto total (FT) encontrada nos dentifrícios distribuídos pelo SUS em Manaus (Figura) está de acordo com a declarada na embalagem de três dos quatro dentifrícios analisados (Tabela 1). A exceção foi o dentifrício C, no qual se esperaria encontrar 1.500 ppm de FT (Tabela 1), mas o valor encontrado foi 82% menor. Em acréscimo ao baixo valor médio encontrado, de 274,1 ppm de FT neste produto, outro aspecto que chamou a atenção foi o alto desvio padrão da média (80%) dos 10 tubos analisados. Isso é facilmente explicado pela diferença da concentração de FT encontrada entre os dois lotes desse dentifrício. Enquanto nos 5 tubos do dentifrício C do lote 11684118 foi encontrada média de 479,6 ppm F de FT, no lote 11681118 foi encontrado apenas 68,4 ppm F de FT. Assim, nos dentifrícios do lote 11684118 foi adicionada uma quantidade de Na_2_FPO_3_ três vezes menor que o declarado na embalagem, enquanto o lote 11684118 sequer foi fluoretado. O valor médio de 68,4 ppm F de FT nos tubos desse lote deve ser atribuído ao fluoreto residual dos ingredientes usados na formulação do mesmo. Assim, esse dentifrício viola a regulamentação brasileira^15^ e do Mercosul^16^, pois o dentifrício não foi formulado como declarado na rotulagem. É admissível, também, o descumprimento do edital de licitação pelo fornecedor do produto ao SUS em Manaus, pois a especificação padronizada para tais produtos é de que o dentifrício deve ser fluoretado na concentração de 1.000 a 1.500 ppm F. Essa flagrante aberração de não encontrar no dentifrício a concentração de FT declarada é rara nos nossos 40 anos de avaliação de dentifrícios do mundo inteiro e só foi verificada para dois dentifrícios de origem chinesa, um que estava sendo vendido no Chile^25^ e outro no Peru^19^. Por outro lado, o mais importante em termos de saúde é quanto do FT de um dentifrício está quimicamente solúvel para ter potencial anticárie^3-5^.

Assim, dos quatro dentifrícios analisados que estavam sendo distribuídos pelo SUS em Manaus, somente o da marca D teria um bom potencial anticárie (Tabela 2), pois apresentou média de 937,9 ppm F de fluoreto solúvel total (FST), muito próximo de 1.000 ppm F, o valor mínimo necessário para ter eficácia anticárie^4^. Em acréscimo, todo o fluoreto desse dentifrício está solúvel (Figura) visto que foi formulado com SiO_2_, um abrasivo compatível com todos os sais de fluoreto, incluindo o Na_2_FPO_3_ usado nessa formulação (Tabela 1). Em contrapartida, a concentração de FST encontrada nos demais dentifrícios foi muito baixa. A baixa concentração de FST encontrada nos dentifrícios A, B e C (Tabela 2) não é apenas explicada por que eles contêm CaCO_3_ como abrasivo (Tabela 1) e este inativa o fluoreto^3,4^, mas também pelo fato que, à exceção do A, estes dentifrícios foram formulados com menos de 1.500 ppm de FT.

Como 57% do FT do A estava insolúvel (Figura), ainda sobraram 642,3 ppm como FST, o que lhe confere um baixo potencial anticárie (Tabela 2). Já o B, além de ter sido formulado com 1.100 ppm F de FT (Tabela 1), 70% desse fluoreto estava insolúvel, restando apenas 339,9 ppm como FST, o que lhe confere um potencial anticárie muito baixo. Comparado ao produto A, a maior porcentagem de Fins no B pode ser explicada pela proximidade desses dentifrícios do prazo de validade (Tabela 1), inclusive o dentifrício de lote 99 da marca B, distribuído na UBS Balbina Mestrinho, estava com o prazo de validade expirado (abril de 2019) há seis meses da coleta. No dentifrício desse lote, dos 1.100 ppm de FT adicionado, apenas 108,9 ppm estavam solúveis (Tabela 2), isto é, 90% do FT dele estava insolúvel, não apresentando efeito anticárie. Esse resultado de diminuição da concentração de FST em dentifrícios formulados com CaCO_3_/Na_2_FPO_3_ de acordo com o tempo de fabricação é bem conhecido da literatura^13^, mas os valores porcentuais encontrados no presente trabalho foram bem maiores.

Por outro lado, o caso do dentifrício C é totalmente atípico, pois a baixíssima concentração média de 42,3 ppm de FST encontrada nos tubos do lote 11681118 é devido ao fato de sequer ter sido fluoretado, o que lhe confere um potencial anticárie nulo. Nos dentifrícios C do lote 11684118, foi encontrada uma média de 324,1 ppm de FST, uma vez que 32% dos 479,7 ppm do FT encontrado estava insolúvel, o que lhe confere um potencial anticárie muito baixo (Tabela 2).

Considerando que dos 99 tubos de dentifrícios analisados somente os oito da marca D podem ser considerados com bom potencial anticárie (Tabela 2), o que representa apenas 8% da amostra, muito dificilmente alguma UBS de Manaus estaria distribuindo para seus usuários só dentifrício de boa qualidade, como de fato mostram os dados da Tabela 3. Assim, se em um extremo havia pessoas recebendo um dentifrício com bom potencial anticárie, caso do D, no outro havia pessoas recebendo um dentifrício sem proteção contra cárie (Nulo), caso do lote 11681118 do dentifrício C. Do mesmo modo, nenhum distrito de saúde da cidade de Manaus fora beneficiado pela qualidade do dentifrício fluoretado distribuído, como demonstrado na Tabela 4.

Esse problema de baixa qualidade do fluoreto de dentifrícios não é exclusividade do Brasil e tem sido relatado em outros países^26,27^. Com exceção das regulamentações dos EUA^28^ e de Madagascar^29^, a maioria das regulamentações mundiais^16,17^ não estabelece que o dentifrício apresente fluoreto solúvel para ter eficácia anticárie, mas recentemente a FDI sinalizou que isso precisa mudar^30^.

Nossos resultados ratificam publicações anteriores^11,18^e mostram claramente a necessidade de revisão da regulamentação brasileira resolução ANVISA RDC Nº 530^15^ para garantir que, principalmente a população assistida pelo SUS, receba dentifrício fluoretado com concentração mínima de fluoreto solúvel com potencial anticárie. Como o Brasil faz parte do MERCOSUL, pode haver interesses multilaterais que precisam ser acordados entre os países desse mercado comum para mudanças nas legislações. Enquanto isso não ocorre, a alternativa seria os gestores do SUS elaborarem editais de licitação em que conste^11^:

1. Que o dentifrício não contenha mais que 1.500 ppm (mg/kg) de fluoreto total (FT);
2. Que pelo menos 1.000 ppm do FT esteja quimicamente solúvel (FST) na amostra fresca (recém-fabricada);
3. Que o dentifrício mantenha pelo menos 800 ppm de fluoreto quimicamente solúvel (FST) pelo prazo de 2 anos de fabricação do produto.

A razão para o máximo de 1.500 ppm de FT é por questão de segurança de produto de livre venda, a de 1.000 ppm de fluoreto solúvel é para garantir um efeito mínimo anticárie e 800 ppm solúvel por 2 anos é por ser uma concentração factível de ser obtida^13^ até pelos pequenos produtores brasileiros de creme dental, não os alijando da competição com as multinacionais, mesmo porque, são eles que ganham as licitações feitas pelo sistema público de saúde brasileiro^11^.

## CONCLUSÃO

Considerando os problemas graves de quantidade e qualidade do fluoreto nos dentifrícios distribuídos pelo SUS em Manaus, a conclusão principal deste trabalho é que a revisão da resolução ANVISA RDC Nº 530 e das regulamentações mundiais garantiria que não só os brasileiros, mas também cidadãos de outros países em desenvolvimento, não mais corressem o risco de usar um dentifrício fluoretado potencialmente ineficaz no controle da cárie dentária.
